# Controllability of the gene regulatory network in zebrafish embryogenesis

**DOI:** 10.1038/s41598-025-33869-9

**Published:** 2025-12-28

**Authors:** Jiyeon Park, Kanghee Cho, Jihwan Lee, Junil Kim

**Affiliations:** 1 Department of Bioinformatics, Soongsil University, 369 Sangdo-ro, Dongjak-gu, Seoul, 06978 Republic of Korea; 2 School of Systems Biomedical Science, Soongsil University, 369 Sangdo-ro, Dongjak-gu, Seoul, 06978 Republic of Korea

**Keywords:** Network controllability, Gene regulatory network, Single cell RNAseq, Zebrafish embryogenesis, Biotechnology, Computational biology and bioinformatics, Developmental biology, Molecular biology

## Abstract

The development in multicellular organisms is governed by gene regulatory networks (GRNs), which are often difficult to control by the theory of network controllability. While transcription factors (TFs) have been studied, the controllability of the entire developmental process, from early embryo to terminal differentiation, remains unclear. To address this, we reconstructed inferred GRNs across developmental stages using single-cell RNA sequencing data from zebrafish embryogenesis. We inferred networks for both trunk and branch trajectories, and calculated the fraction of driver nodes for each. About 80% of nodes were driver nodes in most networks. Notably, the fraction of driver nodes was significantly lower at the final developmental phase than in earlier phases, suggesting that inferred GRNs become more controllable as development progresses. Furthermore, we identified critical TFs—defined as transcription factors originating from critical edges—that may highlight key regulatory points.

**Supplementary Information:**

The online version contains supplementary material available at 10.1038/s41598-025-33869-9.

## Introduction

In multicellular organisms, the developmental process begins with a single cell, the zygote, which differentiates into a wide variety of specialized cell types^1^. This orchestration occurs step by step, with specific genes being activated or repressed at defined time points^2^. These temporal transcriptional changes are harmonized by gene regulatory networks (GRNs), complex systems composed of interacting genes and transcription factors (TFs)^1,3^. Given their central role in development, controlling GRNs, that is influencing or directing their behavior, has long been a critical objective in systems biology and regenerative medicine^4–6^. The ability to steer the transcriptional program of a cell has the potential to reprogram its identity, reverse differentiation, or redirect its fate^7^. However, it remains unclear which genes should be controlled and which GRNs are better controllable.

One of the most notable successes in the area is the induction of induced pluripotent stem cells (iPSCs) by a small set of TFs known as the Yamanaka factors^7^ (*Oct3/4*, *Sox2*, *Klf4*, and *c-Myc*). This approach demonstrated that manipulating a few key genes can reprogram differentiated somatic cells back into a pluripotent state. In the context of network theory, this can be viewed as a practical example of successfully controlling a biological system by perturbing only a subset of nodes within a GRN^8–11^. Although the reprogramming succeeded with only 4–5 TFs, achieving desired cell states remains challenging due to the incomplete understanding of the underlying gene regulatory mechanism.

Network controllability refers to the ability to drive some nodes in a network to a desired state within a finite time through external inputs. Liu et al.^12^ introduced a framework for analyzing the controllability of complex networks by identifying driver nodes as the minimal set of nodes that must receive an external input to control the entire network’s state transitions. They further defined the minimum number of driver nodes (*N*_D_) required to steer a network from any initial state to any desired final state, meaning that a smaller *N*_D_ indicates a network that is easier to control.

Liu et al.^12^ reported that it was found that biological networks such as GRNs are more difficult to control, requiring high *N*_D_ values, when this framework was applied. This suggests structural complexity and robustness in biological systems that differentiate from engineered networks.

To explore how controllability of GRNs changes through development, it is essential to reconstruct inferred GRNs at multiple phases of the developmental trajectory. Traditional bulk transcriptomic approaches measure gene expression as an average across heterogeneous cell populations, making it difficult to capture the transcriptional dynamics of individual cells. In contrast, single-cell RNA sequencing (scRNA-seq) enables high-resolution profiling of gene expression at the individual cell level, facilitating the identification of heterogeneity in transcriptional profiles and the reconstruction of cell fate trajectories^13–15^. By leveraging scRNA-seq data, we can capture transcriptional heterogeneity as well as infer pseudo-temporal dynamics and lineage bifurcations. These characteristics make scRNA-seq data especially advantageous for reconstructing phase-specific inferred GRNs and conducting controllability analyses.

In this study, we reconstructed inferred GRNs at 4 key developmental phases using scRNA-seq data from zebrafish embryos^16^. We then evaluated the structural controllability of each network based on the fraction of required driver nodes. Notably, our analysis revealed that the inferred GRNs in later developmental phases are more controllable, while those in earlier phases exhibit higher complexity and resistance to control. These findings suggest that controllability may serve as a novel metric for understanding the dynamics of cell fate commitment.

## Results

### Reconstruction of zebrafish developmental networks using scRNA-seq

**Fig. 1:**
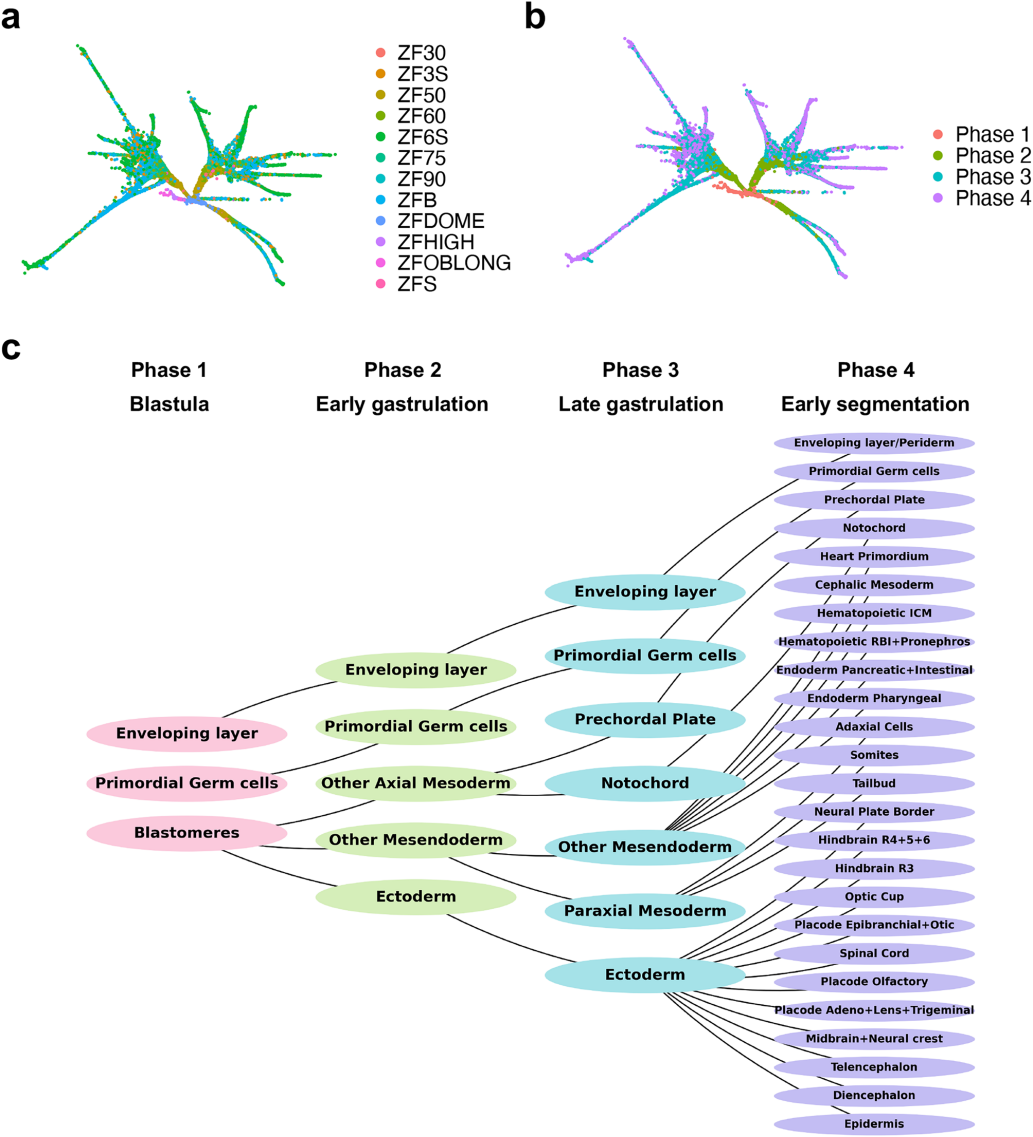
Reconstruction of a developmental process of zebrafish gene regulatory networks. **a** Force-directed layout 2D visualization of early zebrafish embryogenesis. Total 29,036 cells were made up of developmental processes, divided into 4 Phases according to the zebrafish developmental stages. **b** Force-directed layout 2D visualization for early zebrafish embryogenesis divided into 12 developmental stages. **c** The connections of cellular trajectories across four developmental phases—blastula (Phase 1, pink), early gastrulation (Phase 2, green), late gastrulation (Phase 3, blue), and early segmentation (Phase 4, purple). Each node represents a cell type–specific gene regulatory network, and edges indicate inferred developmental transitions between consecutive phases.

To analyze the network controllability at decision-making or bifurcation stages in the developmental trajectory, we reconstructed cell type-specific inferred GRNs using a high-temporal-resolution single-cell transcriptomic dataset from zebrafish embryogenesis. Farrell et al. profiled 38,731 cells across 12 developmental stages of zebrafish embryogenesis^16^. The dataset spans developmental stages from the high blastula stage to morphogenetically distinct stages of differentiation (Fig. 1a). Among the total 38,731 cells, we selected 29,036 annotated cells grouped by 15 trunk and 25 tip cell types and selected 1883 highly variable genes. To systematically examine gene regulatory changes across developmental transitions, we categorized the 12 developmental stages into 4 distinct phases based on major developmental milestones (Fig. 1b)^17^. Phase 1 (high, oblong, dome) represents the Blastula stage; Phase 2 (30–60% epiboly) marks the Early Gastrulation stage; Phase 3 (75% epiboly, 90% epiboly, and bud) corresponds to the Late Gastrulation to Bud stage. These three phases constitute the trunk of the developmental trajectory, representing key transition moments. Phase 4 (3-somites and 6-somites) represents the tip of the trajectory map, where segmentation and organogenesis become more defined. Based on the expression data and pseudotime information of the 40 developmental trajectory^16^, we reconstructed inferred GRNs corresponding to each of 40 cell types using TENET^18^ (Transfer Entropy-based causal gene NETwork), a pseudotime-based GRNs inference method. Figure 1c illustrates the connections of the cellular trajectories across developmental phases (Phases 1–4). We obtained the TF list comprised 2,036 genes from AnimalTFDB 4.0^19^. Quantification of node and edge overlap revealed that GRNs are not independent entities but subgraphs of a larger regulatory network, with higher similarity observed within the same phase and trajectory (Supplementary Figs. 1a–d).

### TENET inferred GRNs include known lineage markers

To assess the biological relevance of the TENET-derived inferred GRNs, we examined the top 10 outdegree hub genes for each network within its trajectory and developmental phase (Supplementary Table S1). In Phase 1 (blastula stage), the Blastomeres network recovered *sall4*^20^ as a high outdegree hub gene, consistent with pluripotency/germline programs that dominate the earliest embryo. Within the ectoderm trajectory, Phase 2 prioritized *sox2*^21^ and *foxd3*^22^, reflecting maintenance of neural progenitor identity (SoxB1) and neural crest multipotency during early gastrulation. By Phase 3 (late gastrulation), *neurog1*^23^ emerged among the top hub genes, marking the onset of neurogenic differentiation.

We applied the same hub-ranking analysis to Phase 4 (early segmentation). Among previously reported zebrafish markers, the Placode Adeno + Lens + Trigeminal network recovered *pitx3*^16^ as a high outdegree hub gene—consistent with its role in lens/cranial placode programs—and the Telencephalon network recovered *emx3*^16^, a telencephalic patterning factor. Across ectodermal lineages in Phase 4, several expected regulators emerged as hub genes: *sox11a*/*sox11b*^24^, which promote neuronal differentiation; *sox2*/*sox3*^21^, which maintain neural progenitor identity; and *zic2a*/*zic2b*^25^, which contribute to neural tube/forebrain patterning. Tissue-specific hub genes were also consistent with known roles: in Hindbrain R4 + 5 + 6, *mafba*^26^, a rhombomere patterning factor; in Optic Cup, *pax6a*^27^, a canonical retina/forebrain regulator; in Placode Adeno + Lens + Trigeminal, *six1b*^28^, a cranial placode determinant; in Placode Epibranchial + Optic, *foxi1*^29^, an otic/epibranchial placode TF; in Spinal Cord, *olig2*^30^, specifying motor-neuron/oligodendrocyte lineages; and in Heart Primordium, *hand2*^31^, a lateral-plate/cardiac regulator. Collectively, these Phase-4 hub genes align with lineage-appropriate developmental functions, supporting the biological relevance of the TENET-derived networks.

### The controllability of 40 zebrafish’s developmental gene regulatory networks

To investigate the controllability of the 40 reconstructed gene regulatory networks (GRNs), we calculated the fraction of driver nodes (*n*_D_) in each cell type’s network. Across the early and mid-developmental phases, our inferred GRNs exhibited consistently high *n*_D_ values (Table 1), suggesting that these networks were structurally difficult to control. This observation is consistent with previous findings by Liu et al.^12^, who reported that biological networks are generally difficult to control and typically exhibit a high *n*_D_, approximately 80%.

**Table 1:** Properties of 40 gene regulatory networks (GRNs) reconstructed from zebrafish developmental cell types.

Phase	GRNs	*N*	*L*	*n*_D_	*l*_r_	*l*_o_	*l*_c_	*D*	*H*	γ
1	Enveloping Layer	503	2711	0.862823	0.0217632	0.977868	0.00036887	5.38966203	1.41985	2.20982081
	Primordial Germ cells	259	954	0.826255	0.0303983	0.964361	0.00524109	3.68339768	1.235732	2.17165245
	Blastomeres	511	1270	0.882583	0.313386	0.677953	0.00866142	2.4853229	1.423221	2.23017674
2	Enveloping Layer	529	1743	0.865785	0.35743	0.627653	0.0149168	3.29489603	1.447612	2.22345159
	Primordial Germ cells	341	641	0.859238	0.218409	0.76131	0.0202808	1.8797654	1.273093	2.41007383
	Ectoderm	451	1130	0.873614	0.311504	0.680531	0.0079646	2.50554324	1.399564	2.23809286
	Other Mesendoderm	473	843	0.892178	0.213523	0.772242	0.0142349	1.78224101	1.306278	2.2194016
	Other Axial Mesoderm	480	1257	0.85625	0.358791	0.633254	0.00795545	2.61875	1.42566	2.25256201
3	Enveloping Layer	584	1156	0.868151	0.262976	0.723183	0.0138408	1.97945205	1.338638	2.39407086
	Primordial Germ cells	397	1208	0.798489	0.234272	0.754139	0.0115894	3.04282116	1.318965	2.26719939
	Ectoderm	436	1238	0.866973	0.214863	0.775444	0.00969305	2.83944954	1.413963	2.20445767
	Paraxial Mesoderm	457	814	0.899344	0.14742	0.837838	0.014742	1.78118162	1.288964	2.14650461
	Other Mesendoderm	477	1009	0.890985	0.278494	0.705649	0.0158573	2.11530398	1.379758	2.35570319
	Notochord	447	749	0.87472	0.245661	0.735648	0.0186916	1.67561521	1.272235	2.20355315
	Prechordal Plate	454	657	0.914097	0.161339	0.83105	0.00761035	1.44713656	1.230409	2.05510966
4	Enveloping Layer	544	2302	0.841912	0.0438749	0.953519	0.00260643	4.23161765	1.290836	2.29417041
	Primordial Germ cells	324	1402	0.746914	0.0763195	0.914408	0.00927247	4.32716049	1.227669	2.3672417
	Spinal Cord	337	699	0.762611	0.374821	0.579399	0.0457797	2.07418398	1.266472	2.54612079
	Diencephalon	319	639	0.821317	0.261346	0.707355	0.0312989	2.0031348	1.28039	2.28520814
	Optic Cup	270	793	0.811111	0.32913	0.655738	0.0151324	2.93703704	1.381626	2.18875359
	midbrain + neural crest	284	554	0.799296	0.279783	0.691336	0.0288809	1.95070423	1.24442	2.44389368
	Hindbrain R3	266	1058	0.736842	0.113422	0.881853	0.0047259	3.97744361	1.292785	2.29394744
	Hindbrain R4 + 5 + 6	316	726	0.803797	0.34573	0.628099	0.0261708	2.29746835	1.310362	2.46964724
	Telencephalon	283	1091	0.75265	0.159487	0.835014	0.00549954	3.85512367	1.347187	2.26629625
	Epidermis	365	757	0.873973	0.245707	0.742404	0.011889	2.0739726	1.356486	2.15727157
	Neural Plate Border	333	1092	0.777778	0.391026	0.60348	0.00549451	3.27927928	1.37895	2.19454937
	Placode Adeno + Lens + Trigeminal	325	1534	0.710769	0.507823	0.481095	0.0110821	4.72	1.349381	2.23508351
	Placode Epibranchial + Otic	356	889	0.772472	0.419573	0.55793	0.0224972	2.49719101	1.319675	2.36222696
	Placode Olfactory	346	1432	0.702312	0.369413	0.618017	0.0125698	4.13872832	1.276427	2.41107073
	Tailbud	299	528	0.879599	0.179924	0.797348	0.0227273	1.76588629	1.28214	2.19869675
	Adaxial Cells	332	498	0.873494	0.154618	0.825301	0.0200803	1.5	1.217218	2.38408741
	Somites	346	1780	0.739884	0.441011	0.553371	0.00561798	5.14450867	1.37596	2.23017205
	Hematopoietic ICM	283	684	0.756184	0.406433	0.562865	0.0307018	2.41696113	1.276187	2.41166907
	Hematopoietic RBI + Pronephros	349	1232	0.679083	0.468344	0.511364	0.0202922	3.53008596	1.271202	2.46907124
	Endoderm Pharyngeal	392	1694	0.714286	0.603306	0.374262	0.0224321	4.32142857	1.355556	2.31163522
	Endoderm Pancreatic + Intestinal	394	1009	0.779188	0.453915	0.508424	0.037661	2.56091371	1.324395	2.39073542
	Heart Primordium	342	1770	0.72807	0.173446	0.824859	0.00169492	5.1754386	1.371556	2.22238996
	Cephalic Mesoderm	327	2028	0.715596	0.231262	0.764793	0.00394477	6.20183486	1.330649	2.17679476
	Prechordal Plate	340	465	0.888235	0.0989247	0.87957	0.0215054	1.36764706	1.174788	2.28500226
	Notochord	356	1427	0.803371	0.170287	0.82691	0.00280308	4.00842697	1.362417	2.19148314

*N* Number of nodes, *L* Number of edges, *n*_D_ Fraction of driver nodes, *l*_r_ Fraction of redundant edges, *l*_o_ Fraction of ordinary edges, *l*_c_ Fraction of critical edges, *D* Network density, *H* network heterogeneity, γ degree exponent.

**Fig. 2:**
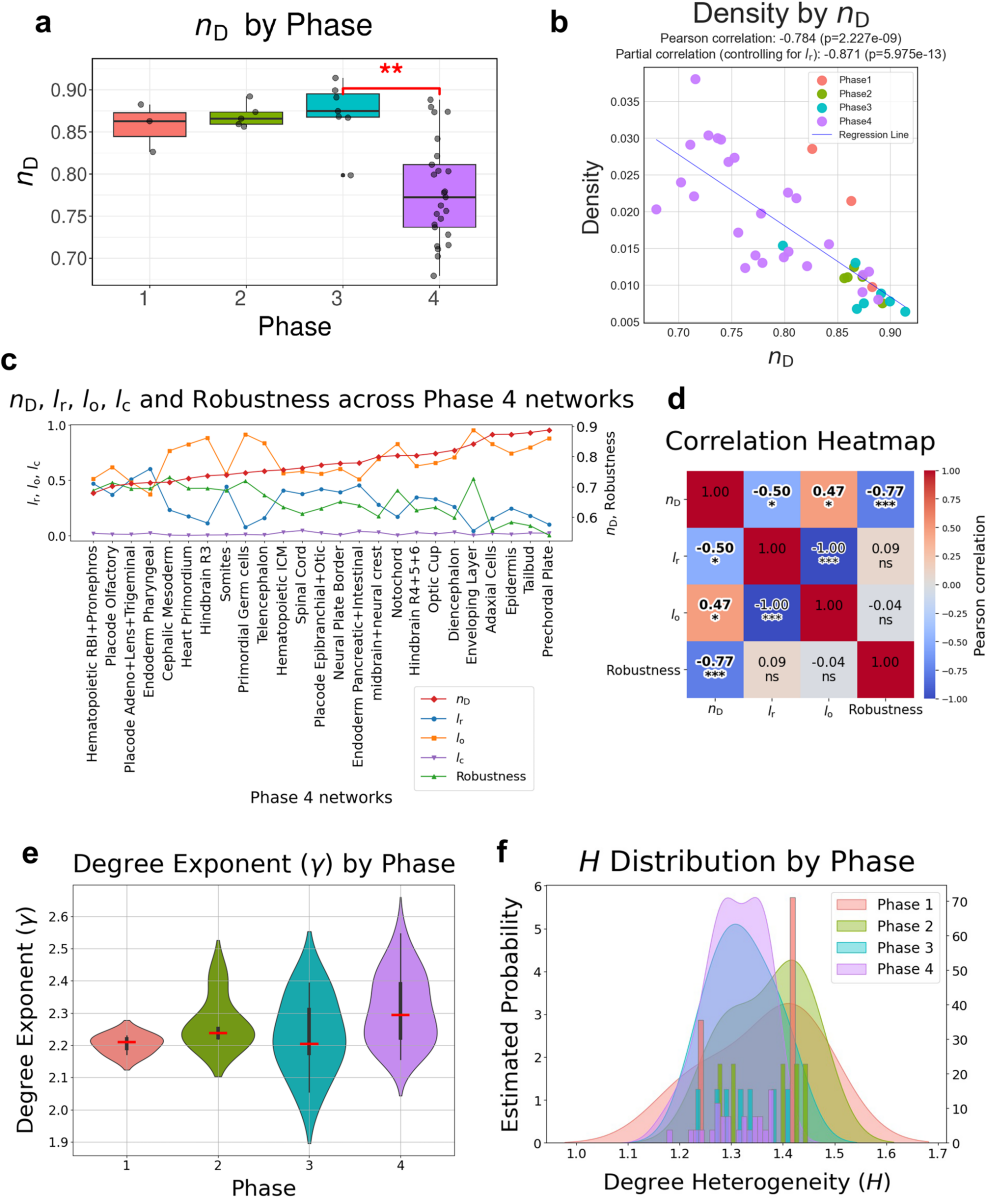
Network properties of 40 cell type-specific gene regulatory networks across 4 developmental phases. **a** Box plot showing the statistically difference in fraction of driver nodes (*n*_D_) among four developmental phases: Red, blastula (Phase 1); Green, early gastrulation (Phase 2); Blue, late gastrulation (Phase 3); and Purple, early segmentation (Phase 4). Kruskal–Wallis rank sum test revealed a significant difference among phases (*χ*^2^ = 16.155, *p* = 0.0011). The Dunn’s post hoc test with Bonferroni correction was performed to evaluate which groups are significantly different (*p* < 0.05). **b** Correlation and partial correlation plot between network density and *n*_D_, controlling for *l*_r_. The Pearson correlation coefficient is – 0.784 (*p* = $2.227$ × $1{0}^{ -9}$) and the partial correlation coefficient is -0.871 (*p* = $5.975$ × ${10}^{-13}$). Each point represents a single network, with colors indicating developmental phases as in (**a**). The blue line represents the linear regression fit. **c** Line plots show the proportions of redundant edges (*l*_r_, blue), ordinary edges (*l*_o_, orange), and critical edges (*l*_c_, purple) on the left y-axis, and the fraction of driver nodes (*n*_D_, red) together with network robustness (green) on the right y-axis across Phase 4 networks. Networks are ordered from left to right by ascending *n*_D_ values. Robustness represents network stability under random edge failure. **d** Correlation heatmap showing pairwise Pearson correlations among the fraction of driver nodes (*n*_D_), edge categories (*l*_r_, *l*_o_), and network robustness, calculated across Phase 4 networks. Color intensity indicates the correlation strength, with blue and red representing negative and positive correlations, respectively. Asterisks denote significance levels (*p < 0.05, **p < 0.01, ***p < 0.001). **e** Violin plots showing the distribution of degree exponent (γ) across the four developmental phases. Colors correspond to phase classifications as shown in (**a**). **f** Distribution of degree heterogeneity (*H*) across developmental phases. Kernel density estimates illustrate the probability density of *H* values for four phases, with each color representing a different phase, as shown in (**a**).

Networks were grouped based on their developmental trajectories, and we assessed whether there were significant differences in the *n*_D_ across these groups. No significant differences were observed when grouped by trajectory (Supplementary Figs. 2a, b). However, when we grouped the networks according to developmental phases, we found a significant difference in the *n*_D_ between Phase 3 and Phase 4. Specifically, Phase 4 networks exhibited a significantly lower *n*_D_ (Fig. 2a). This suggests that the inferred GRNs in Phase 4 are the easiest to control.

Next, we calculated network metrics for each of the 40 inferred GRNs reconstructed from zebrafish embryogenesis to investigate the underlying basis of the differential controllability observed across the developmental phases. In this analysis, edges were classified according to the definitions by Liu et al.^12^: critical edges, whose removal increases the number of driver nodes (*n*_D_) required for full control; redundant edges, which can be removed without affecting the driver node set; and ordinary edges, which do not fall into either of the above categories and may influence the driver node set upon removal. We then computed the fraction of critical (*l*_c_), redundant (*l*_r_), and ordinary (*l*_o_) edges for each network (Table 1). The proportions of these edge types did not show significant differences across developmental phases (Supplementary Figs. 3a–c).

To explore the overall association between network density, edge composition, and controllability (Table 1), we analyzed the relationship between *D* and *n*_D_ and observed a strong negative correlation (*r* = – 0.784, *p* = 2.23 × 10^–9^), suggesting that denser networks are easier to control (Fig. 2b). Among the fraction of edge types, *l*_r_ was negatively correlated whereas *l*_o_ was positively correlated with *n*_D_ (Supplementary Figs. 4a–c), indicating that networks with a higher proportion of redundant edges were also easier to control. Interestingly, *l*_r_ and *D* are independent of each other (Supplementary Fig. 4d), which means that the two network properties network density and the ratio of redundant edges are independently associated with *n*_D_**.**

In Phase 4, both the *n*_D_ and *D* values exhibited a wider range of distribution compared to other phases (Fig. 2a, b). To further explore this diversity from the perspective of controllability, we sorted the GRNs of this phase by their *n*_D_ in ascending order and analyzed the proportions of different edge types as well as network robustness to edge removal (Fig. 2c). From this analysis, we found that network robustness showed a strong negative correlation with *n*_D_, while *l*_r_ and *l*_o_ were not significantly correlated with robustness (Fig. 2c, d). These findings demonstrate that networks with lower *n*_D_ values are more robust under progressive edge removal, irrespective with the redundancy. When the networks were reordered based on *l*_r_ and *l*_o_ values instead of *n*_D_, there was no clear tendency for anatomically related tissues to cluster together (Supplementary Fig. 5).

Additionally, we examined the γ across developmental phases (Fig. 2e, Table 1). In network theory, the degree exponent (γ) is an indicator of network topology, with values between 2 and 3 typically associated with complex scale-free networks. Our results are consistent with previous studies^32,33^ reporting that biological networks often exhibit such scale-free properties. Furthermore, we examined the *H* across developmental phases (Fig. 2f, Table 1). This measure captures the expected degree difference between two randomly chosen nodes, normalized by the mean degree, and provides insight into the unevenness of the degree distribution. Higher values of *H* indicate greater heterogeneity^34^, reflecting the presence of highly connected hub nodes alongside sparsely connected ones. Consistent with the trend observed for *n*_D_, the distribution of *H* moved toward lower values in Phase 4 compared to earlier phases, implying that the network structure became simpler and less heterogeneous over the phases. These findings may account for the reduced *n*_D_ values observed in Phase 4.

Overall, these results indicate that toward the final developmental phase, inferred GRNs undergo a transition toward denser and less heterogeneous regulatory structure, thereby facilitating network controllability.

### Benchmarking fraction of driver nodes using different species and GRN inference tools.

To further validate the finding that fraction of driver nodes (*n*_D_) decreases in phase 4, we performed statistical testing on GRNs reconstructed using WGCNA^35^ and GRNBoost2^36^ (Supplementary Figs. 6a, b) and different GRN inference conditions (Supplementary Figs. 6c-e) from TENET. In both tools, *n*_D_ was significantly lower in phase 4. Other reconstructed conditions (all genes, 70% cell sampling, different edge threshold 0.03) also exhibited that *n*_D_ was significantly lower in phase 4.

To confirm our results in different species, we conducted the analysis using the mouse dataset^37^. As in the zebrafish analysis, we first divided the 37 inferred GRNs obtained from the mouse gastrulation dataset^37^ into 3 phases. Statistical analysis of the *n*_D_ did not reveal significant differences across phases (Supplementary Fig. 7a). When ranked by n_D_ values, we observed that the inferred GRNs for Gut and Spinal Cord appeared among the lower group in the final phase, although the overall trend differed from that of zebrafish (Supplementary Fig. 7b). However, a negative correlation between *n*_D_ and network density (*D*) was observed (Supplementary Fig. 7c).

### Defining a new category for assessing the functional importance of transcription factors

To examine a new aspect of TFs in terms of controllability, we devised a new measure of TF importance based on the edge types. In the context of network controllability, a critical edge is defined as an edge whose removal leads to a loss of controllability, either by disrupting the system’s structure or by increasing the number of driver nodes (*N*_D_) required to control it. We identified such edges by analyzing changes in controllability upon edge removal and defined the TFs at the source of these edges as “Critical TFs” (Supplementary Tables 2, 3, 4, 5).

**Fig. 3:**
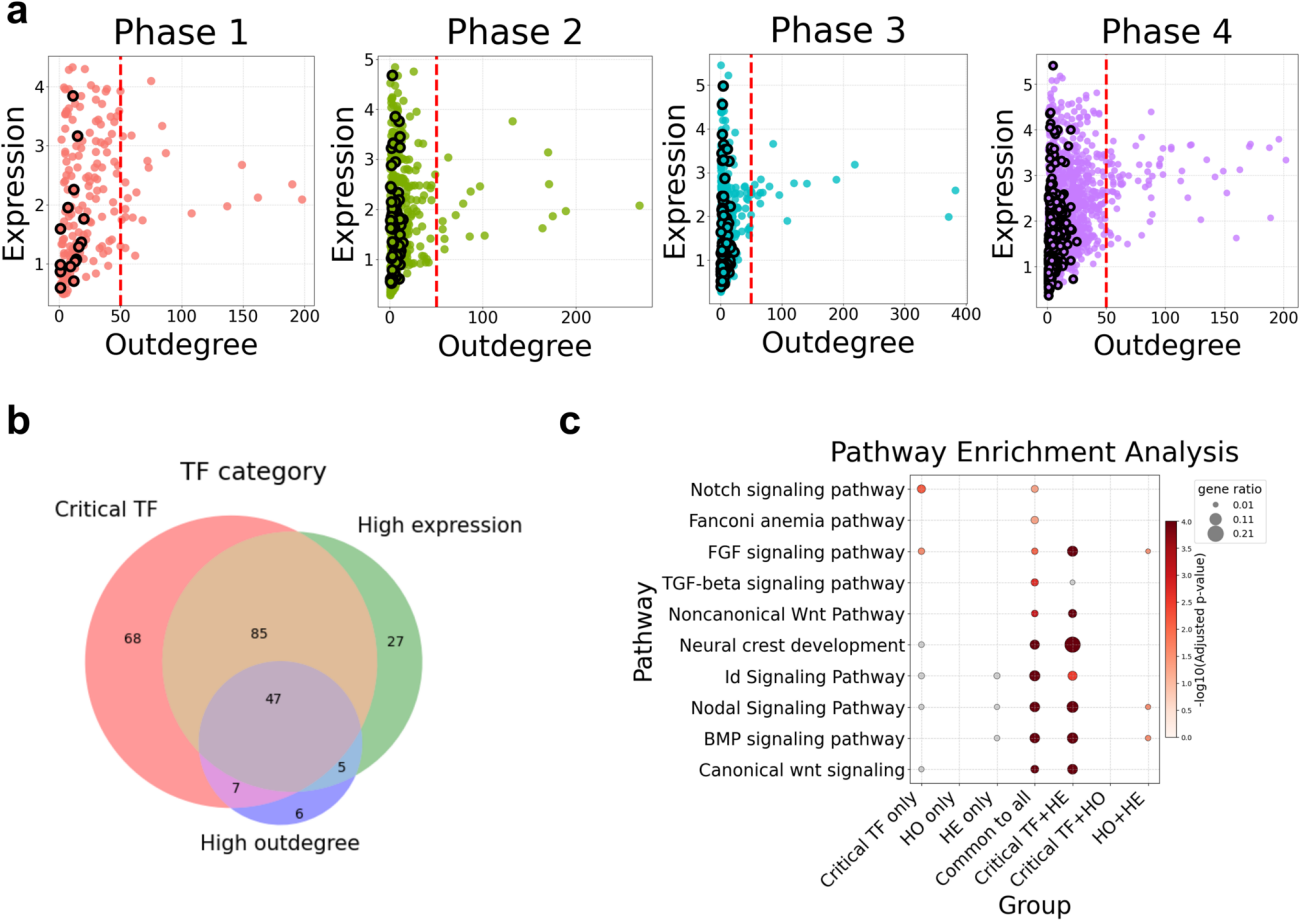
Critical transcription factors (TFs) reveal a unique aspect of TF importance. **a** Scatter plots showing TF categories across four developmental phases (red—Phase 1; green—Phase 2; blue—Phase 3; purple—Phase 4). Normalized expression was obtained by summing raw counts across all cells within each sample, followed by total-count normalization and log1p transformation. Outdegree values are shown for each of the 40 gene regulatory networks (GRNs), with the red dashed line indicating an outdegree threshold of 50. Each dot represents a TF within an inferred GRN, and TFs identified as critical edge sources (Critical TFs) are highlighted with black outlines. **b** Venn diagram integrating TF categories across phases TFs were classified into three groups: Critical TFs (source of a critical edge), high-expression (HE) TFs (expression above the 3rd quantile), and high-outdegree (HO) TFs (outdegree > 50). TFs may belong to multiple groups. **c)** Pathway enrichment analysis of the seven subsets defined by overlaps among the three TF categories. “Critical TF only”, “HO only”, and “HE only” represent subsets unique to each category. “Critical TF + HE”, “Critical TF + HO”, and “HO + HE” indicate intersections between two groups; “Common to all” represents TFs shared among all three categories.

To investigate the features associated with TFs, we examined their outdegree and normalized expression across the 40 reconstructed gene regulatory networks (GRNs), across the developmental phases (Fig. 3a). Although the same TF may appear multiple times across different inferred GRNs or phases, each instance reflects its properties within a specific network context. Interestingly, we found all Critical TFs showed consistently low outdegree values (< 50), suggesting that their regulatory influence may be more selective than other hub TFs.

We next examined how each TF was categorized across all phases and networks. This integration step does not alter any TF’s outdegree; rather, a TF is assigned to a given category if it appeared in that category in *any* of the 40 inferred GRNs. Thus, the integrated summary reflects the union of category membership across the full course of development, rather than properties that occur simultaneously within a single network. The category assignments of each TF within individual GRNs can be inspected in Supplementary Fig. 8. This figure also shows when each TF becomes active during development, allowing us to determine the developmental timing and functional roles associated with each TF category across the entire developmental trajectory. Furthermore, Supplementary Fig. 9 provides a comprehensive visualization of all 40 inferred GRNs, highlighting critical edges, driver nodes, and TF category assignments across developmental phases and trajectories.

Based on this, we classified TFs into three distinct groups based on key regulatory features: (1) Critical TFs, (2) TFs with high outdegree, and (3) highly expressed TFs (Fig. 3b). Among the total TFs, 53 were uniquely identified as Critical TFs, 40 were uniquely categorized as highly expressed, and 7 were specific to the high outdegree group. In addition to these category-specific TFs, 45 TFs were shared among all three categories, while 72 TFs overlapped between the Critical TF and highly expressed categories. This suggests that a substantial proportion of TFs relevant to network controllability also exhibit elevated expression levels.

To further investigate the functional characteristics of TFs in each region of the Venn diagram, we performed pathway enrichment analysis (Fig. 3c). When analyzing TFs that were specific to each individual category, significant pathway enrichment was observed only in the Critical TF group, with the Notch signaling pathway being particularly prominent. In contrast, TFs specific to the high outdegree or highly expressed categories did not show any significant pathway enrichment.

Interestingly, when examining the intersection of Critical TFs and highly expressed TFs or TFs common to all three categories, combinations that included the Critical TF category tended to yield a greater number of significantly enriched pathways. This result suggests that the Critical TF category facilitate identifying pathway-associated gene regulations.

Taken together, these findings suggest that our proposed network-based classification of Critical TF is effective in identifying biologically meaningful signaling pathways. In contrast, traditional classifications based solely on outdegree or expression level are unable to capture such functional specificity, highlighting their limitations^32^.

## Discussion

In this study, we demonstrate the integration of network controllability theory with dynamic single-cell transcriptomic data to explore how regulatory complexity evolves during embryonic development. A long-standing goal in systems biology has been to understand and ultimately guide the behavior of GRNs^4^. In this context, we applied a controllability framework to inferred GRNs reconstructed from early zebrafish development, aiming to derive biologically meaningful interpretations from network structure. We analyzed the structural characteristics of inferred GRNs from the perspective of controllability across developmental phases. One of the most notable findings was observed in Phase 4, where the fraction of driver nodes (*n*_D_) was significantly lower compared to earlier phases, indicating that networks at this phase are more easily controllable.

The observed increase in network controllability in Phase 4 may reflect fundamental changes in cellular organization and regulatory requirements^12,38,39^. In the early developmental phases, cells maintain a pluripotent state, characterized by the potential to differentiate into various lineages^40^. During this period, complex and heterogeneous regulatory networks are necessary to integrate diverse external signals and maintain developmental plasticity^41^, making network control difficult. In contrast, by the final developmental phase, cells have differentiated into specific lineages and established stable cell identities, accordingly, intrinsic regulatory programs prevail and discrete, stabilized state transitions are observed in single-cell studies^42^. As a result, cells increasingly rely on intrinsic regulatory mechanisms rather than external signaling, leading to a more compact and organized network structure. Within controllability theory, such reorganizations tend to lower the minimum fraction of driver nodes (*n*_D_), providing a mechanistic rationale that is consistent with the reduced *n*_D_ we observe in Phase 4^12,38^. These considerations strengthen our interpretation while keeping it aligned with prior GRN architecture frameworks^39^. These findings not only enhance our understanding of developmental progression but also suggest promising applications in biomedical engineering, such as cellular reprogramming or targeted manipulation of differentiation pathways.

Similar phenomena have been observed in engineered networks, where systems with higher density and simpler topologies, such as the backbone structure of the Internet^43^ or optimized electrical grids^44^, exhibit enhanced controllability and robustness. Likewise, the transition of inferred GRNs toward a denser and less heterogeneous network organization during Phase 4 may reflect an evolution toward an optimized regulatory system, facilitating more efficient control with minimal external input.

Interestingly, even within Phase 4, there was notable variability in network controllability across individual inferred GRNs. As *n*_D_ values decreased, networks tended to exhibit a higher fraction of redundant edges (*l*_r_) and a lower fraction of ordinary edges (*l*_o_). The inferred GRNs characterized by low *n*_D_, high *l*_r_, and low *l*_o_ values were not only easier to control from the perspective of control efficiency but also demonstrated greater stability during control attempts—meaning that the system responded reliably without collapsing or becoming unstable. This reflects enhanced control stability.

An increase in *l*_r_ indicates the presence of redundant regulatory paths, suggesting that key functions can be preserved even if specific regulatory routes are disrupted. In this sense, the structural configuration supports robustness in the regulatory system. These findings imply that Phase 4 is not only a stage where control becomes easier overall, but also one in which robustly controllable networks emerge, distinguishing themselves from those that are only controllable but less stable.

The importance of a gene is often inferred from its expression level in each cell type. This also applies to TFs, whose biological relevance is typically assessed based on how strongly they are expressed. In addition to expression levels, the number of downstream genes regulated by a TF, referred to as its outdegree in the inferred GRNs, can also serve as an indicator of its functional significance. For example, if a TF regulates many target genes within the inferred GRNs that is active during a specific biological process, it is likely to play a key regulatory role in that process. Therefore, we further defined Critical TF that is a TF category based on their role as sources of critical edges. This Critical TF category enabled the identification of a significantly enriched pathway Notch signaling, highlighting its utility in capturing functionally important regulatory elements within the inferred GRNs.

Among the TFs classified as Critical TFs, we identified *her12*, *her15.1*, and *her15.2* as being involved in Notch signaling. These genes are homologs of the human *HES5* gene, a canonical target of the Notch signaling pathway and a transcriptional repressor belonging to the basic helix-loop-helix (bHLH) family^45–47^. *HES5* plays a key role in maintaining cells in an undifferentiated or progenitor state by repressing the expression of differentiation-promoting genes^48,49^. It is especially well characterized in the context of neural and cardiac development, where it helps regulate the balance between proliferation and differentiation of progenitor cells^50,51^.

In addition, from the intersection of the three TF categories, we also identified *her6* and *her9* genes involved in Notch signaling and homologous to the human *HES1* gene^45^. Like *HES5*, *HES1* functions as a transcriptional repressor that inhibits differentiation and helps maintain stem or progenitor cell identity^48,49^. The Notch signaling pathway itself is closely associated with cell adhesion signaling and is known to be essential for tissue development and cell fate determination^52–54^.

While the high outdegree and high expression criteria emphasize TF-specific features such as connectivity or transcriptional abundance, the Critical TF criterion reflects network-level regulatory roles, which may be more closely linked to biologically meaningful pathways.

Our study has also several limitations. First, netctrl considers only network topology and does not incorporate edge weights, potentially leading to the loss of quantitative information from GRN inference tools. To address this, we focused on the fraction of driver nodes and on transcription factors associated with critical edges. Second, our findings remain computational predictions, and future experimental validation will be essential to confirm the role of critical TFs and the controllability framework in inferred GRNs. Finally, although zebrafish GRNs exhibited a clear reduction in the fraction of driver nodes (*n*_D_) at the final developmental phase, this pattern was not reproduced in the mouse dataset (E6.5–E8.5, predominantly gastrulation). Several biological and dataset-specific factors likely contribute to this discrepancy. The two datasets capture fundamentally different portions of embryonic development: the zebrafish dataset spans a broad window from late blastula through gastrulation and into early segmentation (approximately 3.3–12 hpf), during which GRNs undergo progressive structural compaction. In contrast, the mouse dataset represents only the gastrulation period, and thus does not include later stages where reductions in nD emerge in zebrafish. As a result, it remains unclear whether mouse GRNs would show similar controllability trends at developmental stages beyond gastrulation. Furthermore, developmental stage labels such as “blastula,” “gastrulation,” and “segmentation” do not represent temporally equivalent states across species. Zebrafish and mouse define developmental progression using distinct morphological and molecular criteria—zebrafish by hours post fertilization, epiboly percentage, and somite counts, and mouse by Theiler stages or embryonic days—which limits direct alignment of GRN dynamics between the two organisms^17,31^. These differences in staging systems, combined with species-specific regulatory kinetics and lineage diversification patterns, likely underlie the inconsistent *n*_D_ patterns observed across taxa. We therefore consider this discrepancy an important limitation of our study and a key motivation for extending cross-species analyses to later developmental stages in mammals.

## Methods

### Data acquisition and preprocessing

We utilized publicly available single-cell RNA sequencing data from Farrell et al.^16^, which profiled the transcriptomes of 38,731 zebrafish embryonic cells spanning 12 developmental stages, from the high blastula stage (3.3 h post-fertilization, hpf) to the 6-somite stage (12hpf). The dataset was generated using Drop-seq, a massively parallel single-cell RNA seq (scRNA-seq) platform that enables transcriptional profiling of thousands of individual cells^55^. We used the author-provided cell-by-gene count matrix and metadata, including stage annotations and segment assignments. And we selected a subset of 29,036 cells, excluding those with missing annotations such as undefined stage or segment information We retained 1883 highly variable genes (HVGs), as defined in the origin study. Variable genes were identified per developmental stage by fitting a null model of technical variability, and the union of stage-specific HVGs was used for downstream analysis. A gene was considered biologically variable if its coefficient of variation exceeded 1.3.5 times the expectation from the null model (based on a negative binomial assumption)^16^. All preprocessing and downstream analyses were conducted in R v4.4.1.

### Phase assignment and trajectory-based segmentation

We categorized the 12 developmental stages into 4 biologically meaningful phases based on their order in embryonic time and developmental progression. The 4 phases corresponded 3, 5, 7, and 25 transcriptionally defined cell types, respectively. Phase 1 (ZFHIGH, ZFOBLONG, ZFDOME) included 3 cell types, corresponding to primordial germ cells, enveloping layer (EVL), and blastomeres. Phase 2 (ZF30, ZF50, ZFS, ZF60) comprised 5 cell types, including ectoderm, other mesendoderm, and other axial mesoderm populations. Phase 3 (ZF75, ZF90, ZFB) contained 7 cell types, reflecting bifurcation events such as the subdivision of axial mesoderm into notochord and prechordal plate, and mesendoderm into paraxial mesoderm, and other mesendoderm. Phase 4 (ZF3S, ZF6S) consisted of 25 terminal cell types, representing fate-committed transcriptional states in the trajectory tips generated by URD, a single cell trajectory reconstruction tool^16^. Cell types were assigned based on the segment and tip annotations provided in the original URD trajectory^16^.

### GRN reconstruction

We reconstructed phase and cell type-specific inferred GRNs using TENET, a pseudotime-based network inference algorithm designed to recover directed regulatory interactions from single-cell transcriptomic data^18^. For each of the 40 cell types defined across the 4 developmental phases, we used the expression matrix (cell x genes), pseudotime vector result, and a list of transcription factors (TFs) as inputs to the TENET. We utilized a curated list of 2036 zebrafish TFs obtained from AnimalTFDB 4.0^19^. The TF list was used to specify potential regulatory genes within the 1883 highly variable genes (HVGs) retained from the expression matrix. While all HVGs were included as potential targets, only genes matching the TF list were considered as candidate regulators. Each TENET run was performed on a subset of cells corresponding to either a trunk or terminal tip cell type, ordered by pseudotime based on the URD object. Directed edges were inferred based on temporal precedence and co-expression patterns.

TENET was implemented using Open MPI v4.1.2, OpenJDK v11.0.26, Python v3.10.12, JPype1 v1.6.0, and statsmodels v0.14.5.

We also reconstructed inferred GRNs with WGCNA^35^ v1.73 and GRNBoost2^36^ implemented in arboreto v0.1.6. Since WGCNA constructs undirected co-expression networks, we assigned arbitrary edge directions prior to analysis. This analysis was performed using the zebrafish dataset with cell sampling. GRNBoost2 was performed using the zebrafish dataset with no cell sampling.

### Quantifying network controllability and topological properties

To evaluate the structural controllability of each reconstructed inferred GRNs, we applied the framework introduced by Liu et al.^12^, which quantifies the minimum number of driver nodes required for full network control. We applied this framework to all 40 cell type-specific inferred GRNs using the package netctrl v0.2.0 (https://github.com/ntamas/netctrl) with igraph v0.10.3 and libxml2 v2.9.13. For each network, we computed the number and fraction of driver nodes (*n*_D_), as well as classified the directed edges according to their contribution to network controllability. Edges were categorized as critical, ordinary, or redundant, based on their effect on the number of required driver nodes. An edge was classified as critical if its removal increased the number of driver nodes. To assess the structural complexity of each inferred GRNs, we calculated the network density (*D*). *D* was defined as the ratio of the number of observed directed edges to the maximum possible number of directed edges between nodes, using the formula:

$$D = \frac{L}{{N\left( {N - 1} \right)}}$$

where *L* is the number of edges and *N* is the number of nodes in the network. We also quantified degree heterogeneity (*H*) using the following formula^34^:

$$H = \frac{\sum_{{k}_{i}}\sum_{{k}_{j}}|{k}_{i}-{k}_{j}| \cdot P({k}_{i})\cdot P({k}_{j})}{<k>}$$

where $P(k)$ denotes the probability that a node has degree $k$, and $<k>$ represents the average degree. To further characterize the scale-freeness of the networks, we estimated the degree exponent γ. For each inferred GRNs, we first calculated the degree distribution *P(k)* and its complementary cumulative distribution function (CCDF) $F\left( k \right) = \mathop \sum \limits_{k^{\prime} = k}^{\infty } P\left( {k^{\prime}} \right)$, which represents the probability that a node has degree at least *k*. We transformed both *k* and *F(k)* into logarithmic scales and performed a linear regression on the resulting *log F(k)* versus $log k$ data using SciPy. To avoid bias from flat regions in the CCDF, only non-redundant values were used for fitting. The regression slope *a* was then used to compute the degree exponent(γ) as the formula^56^:

$$\gamma = 1-a$$

A power-law degree distribution with 2 < γ < 3 is considered a hallmark of scale-free networks, which often exhibit high robustness and sparse connectivity.

### Edge-robustness analysis

Edge-robustness was evaluated for each inferred GRN in the python package NetworkX v3.4.2. A summary metric we tracked is the fraction of nodes contained in the largest weakly connected component (GCC fraction). To simulate random edge failure, 20 independent random permutations of the full edge set were generated for each inferred GRN. Edges were then sequentially removed according to each permutation, and the GCC fraction was recorded as a function of the fraction of edges removed. The 20 runs were aggregated to obtain the mean and standard deviation at each removal fraction. Robustness was quantified as the area under the GCC-edge removal curve (AUC), calculated by trapezoidal integration.

### Pathway enrichment analysis

To identify biological pathways enriched among gene sets of interest, we performed pathway enrichment analysis using Enrichr^57,58^ via its programmatic API interface. Specifically, we queried two zebrafish-compatible databases: KEGG_2019^59^, WikiPathways_2018^60^. Gene lists were submitted to Enrichr using the Python requests module to access the Enrichr REST API (http://maayanlab.cloud/FishEnrichr). For each gene set, we collected pathway terms along with the corresponding adjusted *p* values (Benjamini–Hochberg correction), combined scores, and gene overlap statistics. only pathways with adjusted *p* values < 0.05 were considered significantly enriched.

### Statistics and reproducibility

As the distribution of *n*_D_ was non-normal, we performed a Kruskal–Wallis test, followed by Dunn’s post hoc test to assess statistical significance between phases. To assess differences in the fraction of driver nodes (*n*_D_) across the 4 developmental phases, we performed non-parametric statistical testing. Because the distribution of driver node fractions did not satisfy the assumption of normality (as assessed by Shapiro–wilk test, *p* < 0.05), we used the Kruskal–Wallis test, a non-parametric alternative to one-way ANOVA, to compare the 4 groups. Following the Kruskal–Wallis test, we performed Dunn’s post hoc test with Bonferroni correction for multiple comparisons. All statistical analyses were performed in R v4.2.2 using the stats and FSA packages.

## Supplementary Information

Supplementary Information 1.

Supplementary Information 2.

## Data Availability

The zebrafish embryogenesis scRNAseq data analyzed during this study are included in this published article16 and available in the Gene Expression Omnibus (GEO) with accession number (GSE106587). The mouse data also published in this article37 and available in Accession number E-MTAB-6967. A analysis script and various result datas are available at (https://github.com/kh22cho/single-cell-grn-control).
