# Supplementary material for: Controllability of the gene regulatory network in zebrafish embryogenesis
Source: Sci Rep. 2025 Dec 28;16:3783. doi: 10.1038/s41598-025-33869-9 (PMC12852106; doi:10.1038/s41598-025-33869-9)
Supplement: Supplementary file 2 — Supplementary Information 2. [file 41598_2025_33869_MOESM2_ESM.pdf]

Controllability of the gene regulatory network in zebrafish embryogenesis

Jiyeon Park[1*], Kanghee Cho[1*], Jihwan Lee[2], and Junil Kim[1,2@]

[1]Department of Bioinformatics, Soongsil University, 369 Sangdo-Ro, Dongjak-Gu, Seoul 06978, Republic of Korea, [2]School of Systems Biomedical Science, Soongsil University, 369 Sangdo-Ro, Dongjak-Gu, Seoul 06978, Republic of Korea

* These authors contributed equally as co-first authors.

@To whom correspondence should be addressed to JK (Tel: +82-2-820-0452; E-mail: junilkim@ssu.ac.kr)

Supplementary Figures

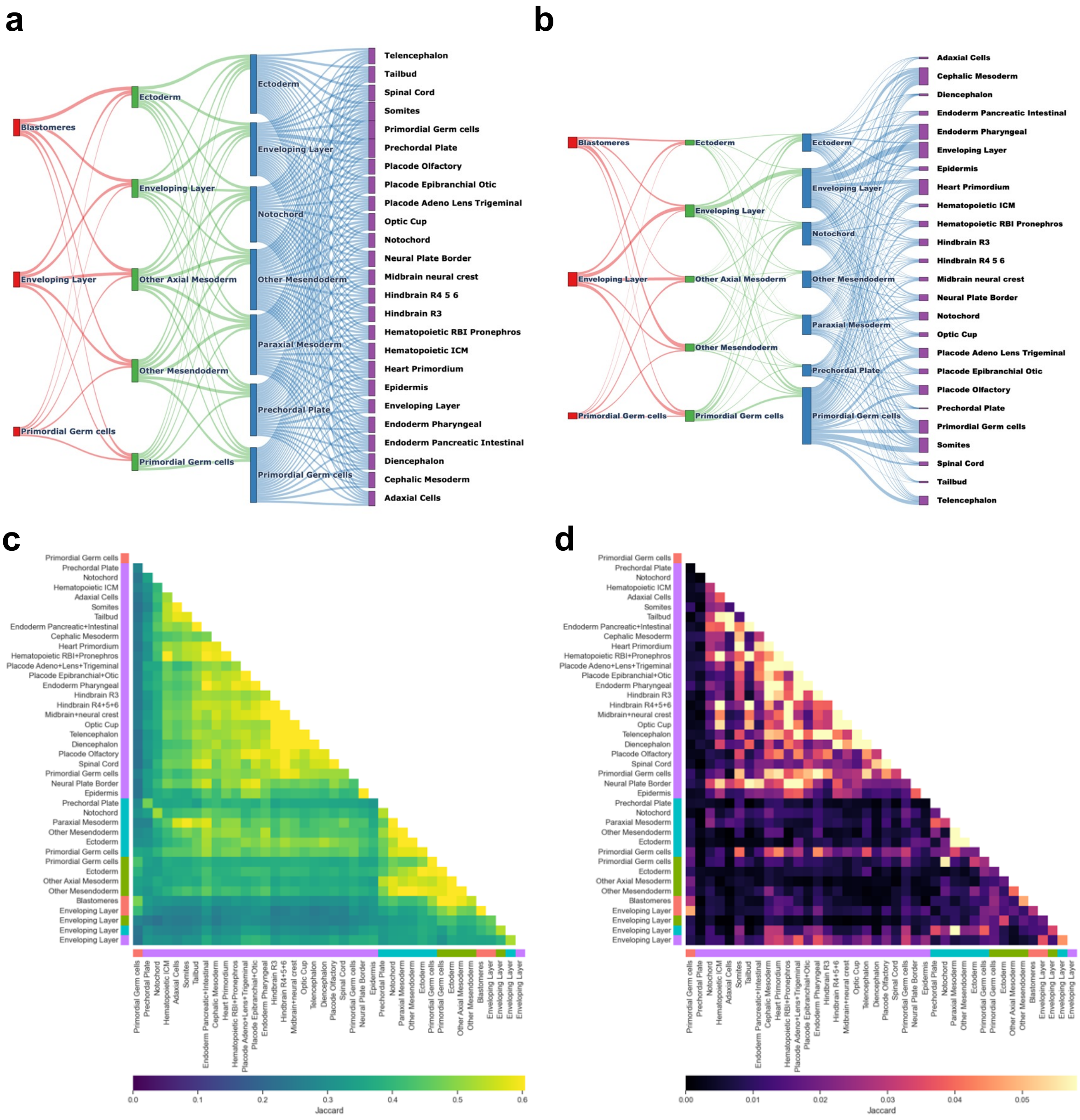

Supplementary Figure 1. Network overlap and independence analysis across inferred GRNs during zebrafish embryogenesis. (**a**) Alluvial plot showing overlapping nodes among the inferred gene regulatory networks (GRNs). (**b**) Alluvial plot showing overlapping edges across the same GRNs. Phase color scheme: red, Phase 1 (blastula); green, Phase 2 (early gastrulation); blue, Phase 3 (late gastrulation); purple, Phase 4 (early segmentation). (**c**) Jaccard index heatmap calculated based on node overlap. (**d**) Jaccard index heatmap calculated based on edge overlap. Phase color scheme follows that used in panels (a–b).

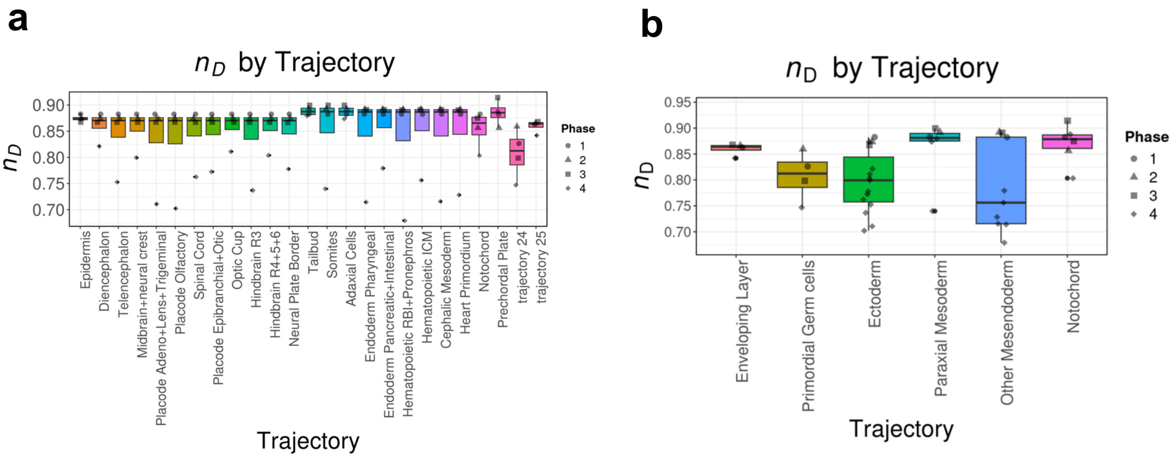

Supplementary Figure 2. Distribution of the fraction of driver nodes (n_D) based on lineage trajectories during zebrafish embryogenesis. (**a**) Distribution of the fraction of driver nodes (n_D) across 25 trajectories during zebrafish embryogenesis ($\chi^2 = 26.674$, $p = 0.3199$). Each box represents a single trajectory, which contains 4 gene regulatory networks (GRNs) corresponding to different developmental phases, selected based on lineage relationships. Each phase is indicated by distinct point shapes: Phase 1, circle; Phase 2, triangle; Phase 3, square; and Phase 4, diamond. (**b**) Distribution of n_D based on trajectories grouped up to Phase 3 (Kruskal-Wallis chi-squared = 11.125, df = 5, p-value = 0.04895). Point shapes correspond to phases as in (a).

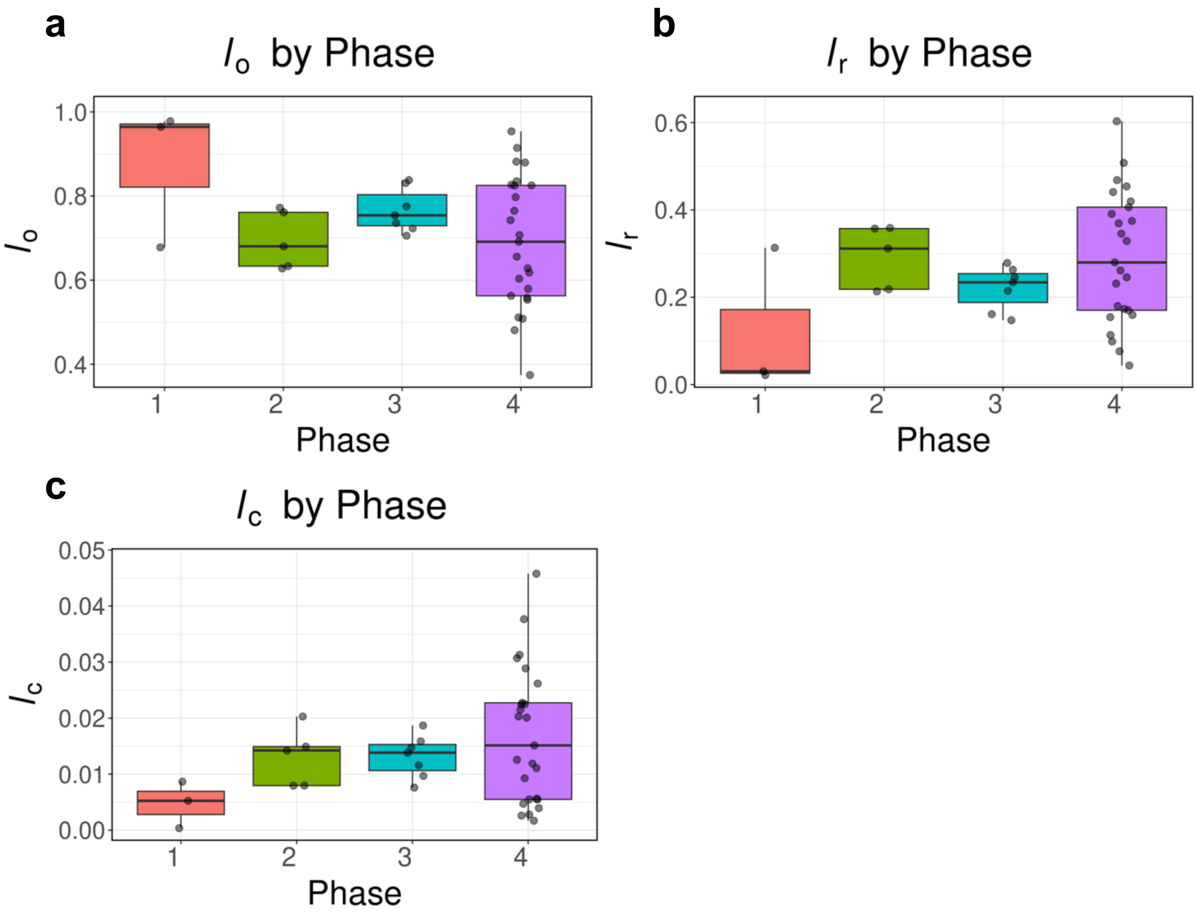

Supplementary Figure 3. Box plots comparing edge types contributing to structural controllability across four zebrafish developmental phases. **(a)** Fraction of ordinary edges (l_o), **(b)** Fraction of redundant edges (l_r), and **(c)** Fraction of critical edges (l_c) calculated for each inferred gene regulatory network (GRN) and compared across developmental phases. Phase color scheme: red, Phase 1 (blastula); green, Phase 2 (early gastrulation); blue, Phase 3 (late gastrulation); purple, Phase 4 (early segmentation). Each point represents an individual inferred GRN. Ordinary edges accounted for largest proportion across all phases, whereas critical edges remained a minor fraction. An increasing trend in l_c was observed Phase 1 to Phase 4. However, these differences were not statistically significant based on Kruskal-Wallis rank sum test ($p > 0.05$).

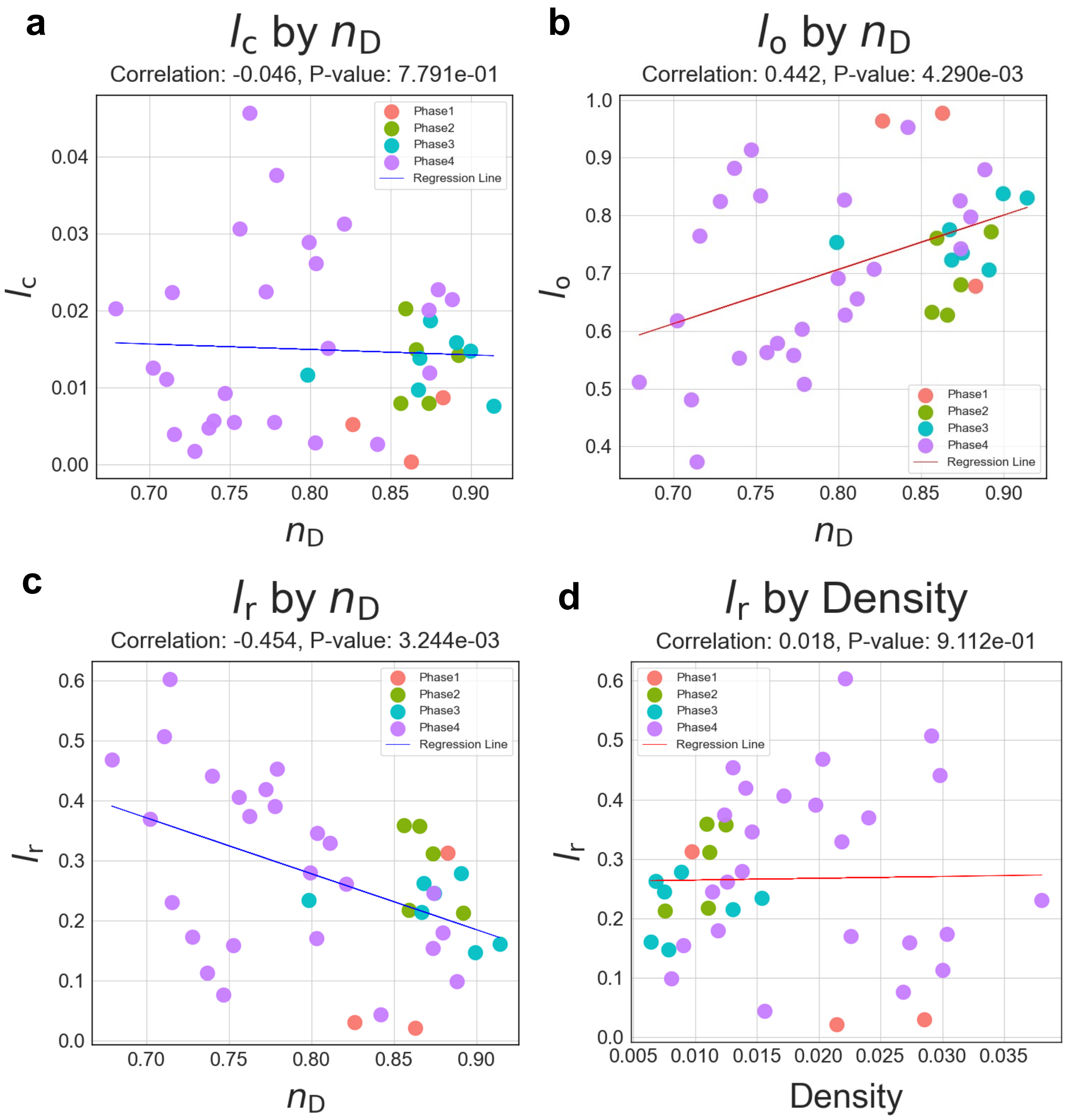

Supplementary Figure 4. Correlation between edge classes and controllability across zebrafish developmental phases. a–c Correlation plots between the fraction of driver nodes (n_D) and each edge class: (**a**) critical edges (l_c); (**b**) ordinary edges (l_o); and (**c**) redundant edges (l_r). (**d**) Correlation plot between network density (D) and redundant edges (l_r). Each dot represents a gene regulatory network (GRN), and solid lines indicate linear regression fits. Phase color scheme: pink, Phase 1 (blastula); green, Phase 2 (early gastrulation); blue, Phase 3 (late gastrulation); purple, Phase 4 (early segmentation).

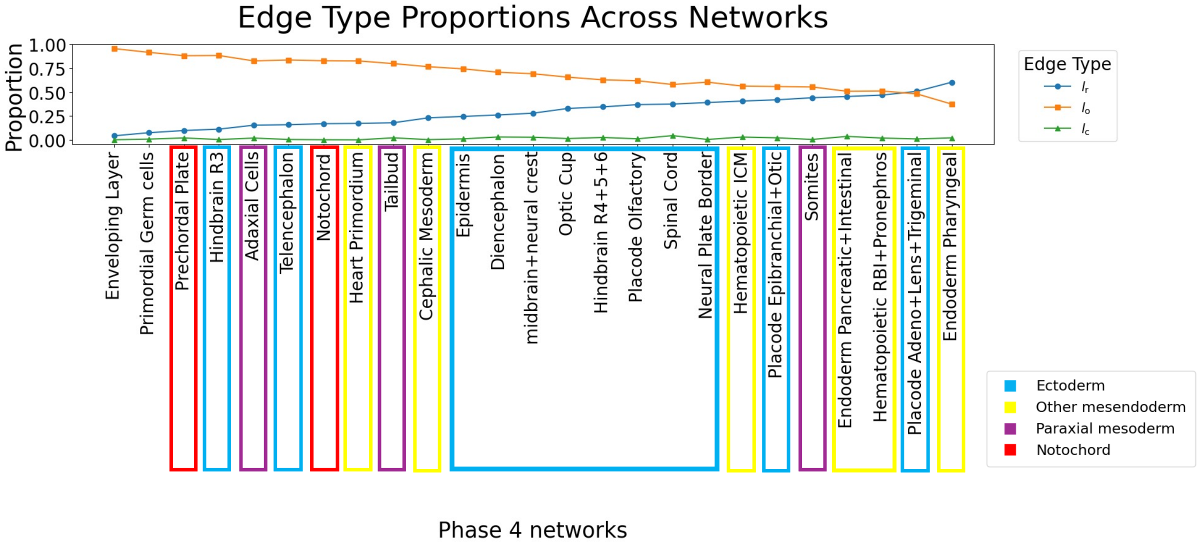

Supplementary Figure 5. Edge type proportions across Phase 4 networks after reordering by redundant-edge fraction (l_r). Line plots show the proportions of redundant (l_r, blue circles), ordinary (l_o, orange triangles), and critical (l_c, green squares) edges for each of the 40 Phase 4 gene regulatory networks (GRNs), ordered by increasing l_r. Colored boxes indicate tissue categories: blue, ectoderm; yellow, other mesendoderm; purple, paraxial mesoderm; and red, notochord.

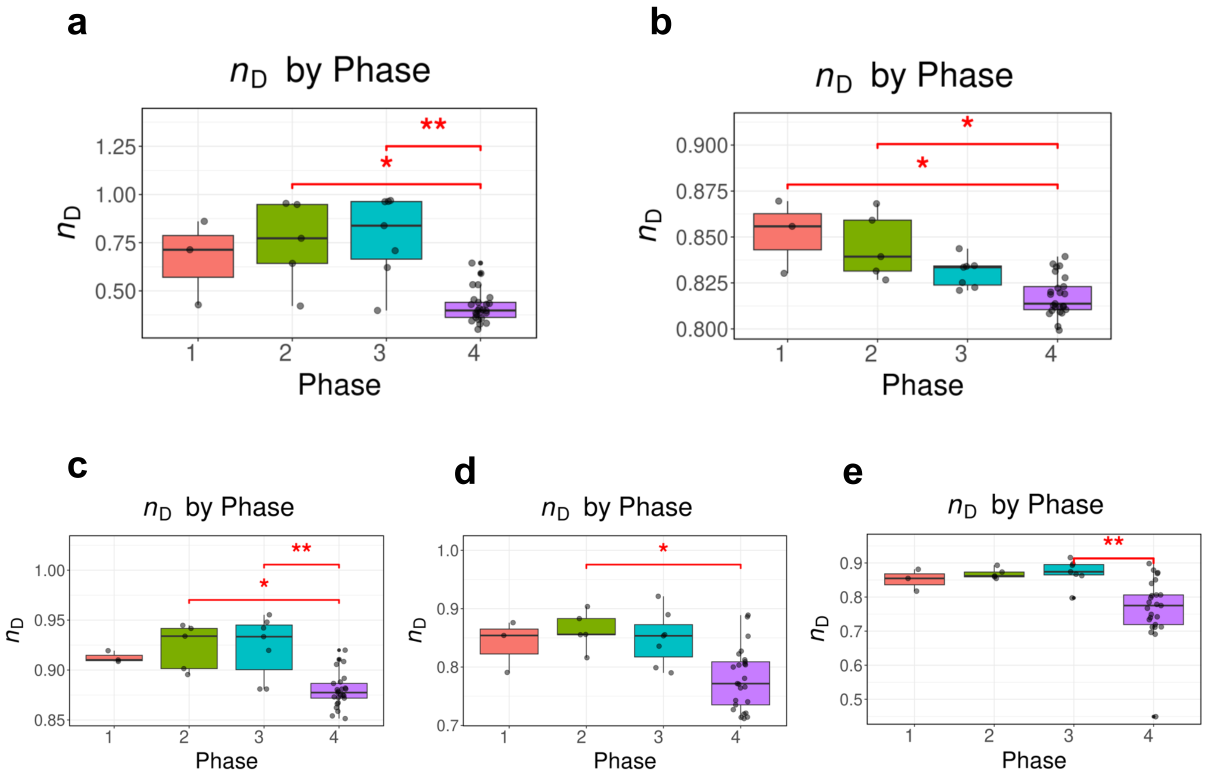

Supplementary Figure 6. Statistical benchmarking of the fraction of driver nodes (n_D) across zebrafish developmental phases using different gene regulatory network inference methods and TENET parameter settings. (a–b) Box plots showing the distribution of n_D from inferred gene regulatory networks (GRNs) reconstructed using different inference tools for benchmarking analysis. **(a)** WGCNA (Kruskal–Wallis χ^2 = 18.346, p = 0.00037); random sampling was performed for cell populations with large cell numbers prior to analysis. **(b)** GRNBoost2 (Kruskal–Wallis χ^2 = 16.757, p = 0.00079); sampling was not applied. **(c–e)** Box plots showing n_D distributions from GRNs inferred with TENET under varying reconstruction parameters: **c** all genes included (Kruskal–Wallis χ^2 = 18.945, p = 0.00028); **(d)** 70% cell sampling (Kruskal–Wallis χ^2 = 14.259, p = 0.00257); € edge threshold = 0.03 (Kruskal–Wallis χ^2 = 15.528, p = 0.00142). Phase color scheme: pink, Phase 1 (blastula); green, Phase 2 (early gastrulation); blue, Phase 3 (late gastrulation); purple, Phase 4 (early segmentation). Each point represents an individual inferred GRN.

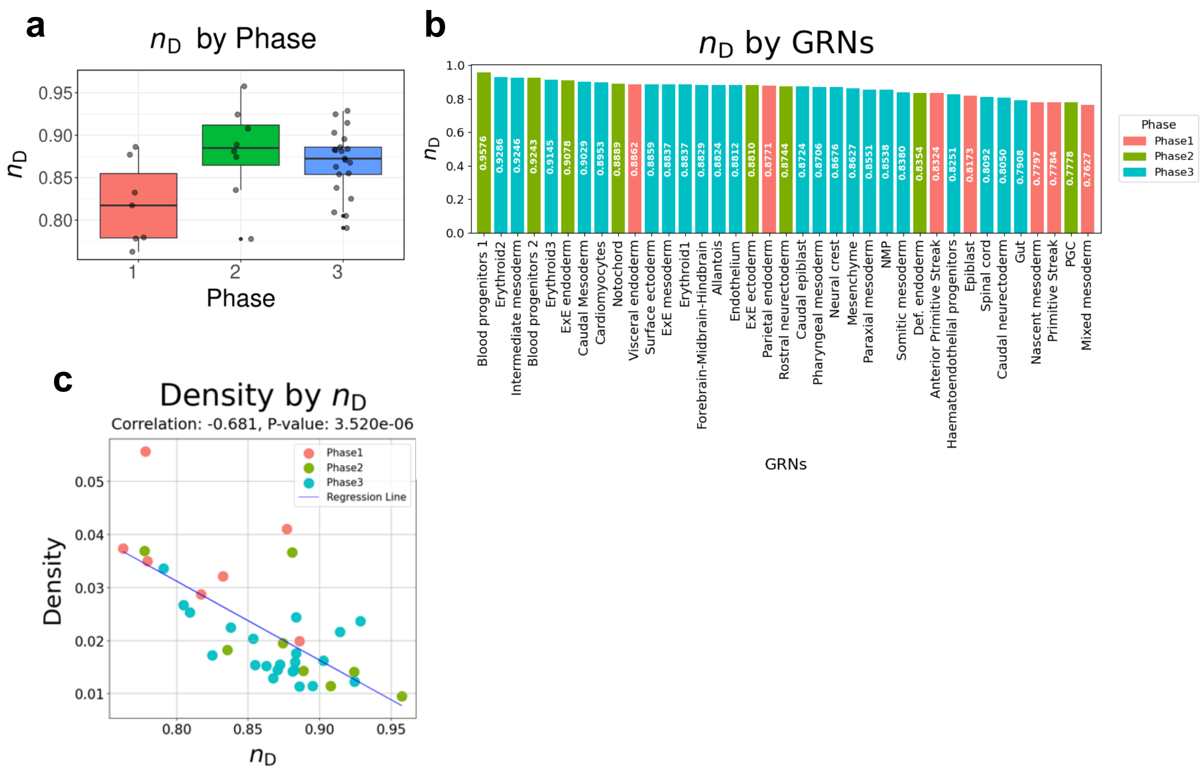

Supplementary Figure 7. Cross-species validation of network controllability using mouse gastrulation data. (**a**) Box plot showing the fraction of driver nodes (n_D) across 37 inferred gene regulatory networks (GRNs) from mouse gastrulation data (Kruskal–Wallis $\chi^2 = 5.572$, $p = 0.0617$). Colors indicate developmental phases: red, early; green, middle; blue, late. (**b**) Bar plot showing n_D values for individual mouse GRNs. White numbers denote the n_D value of each inferred GRN, and bar colors correspond to the same phase scheme as in (**a**). (**c**) Correlation between n_D and network density (*D*) in mouse GRNs, showing a negative relationship consistent with zebrafish results. Each point represents a single inferred GRN, and the blue line indicates the linear regression fit.

TF category distribution across networks by phase

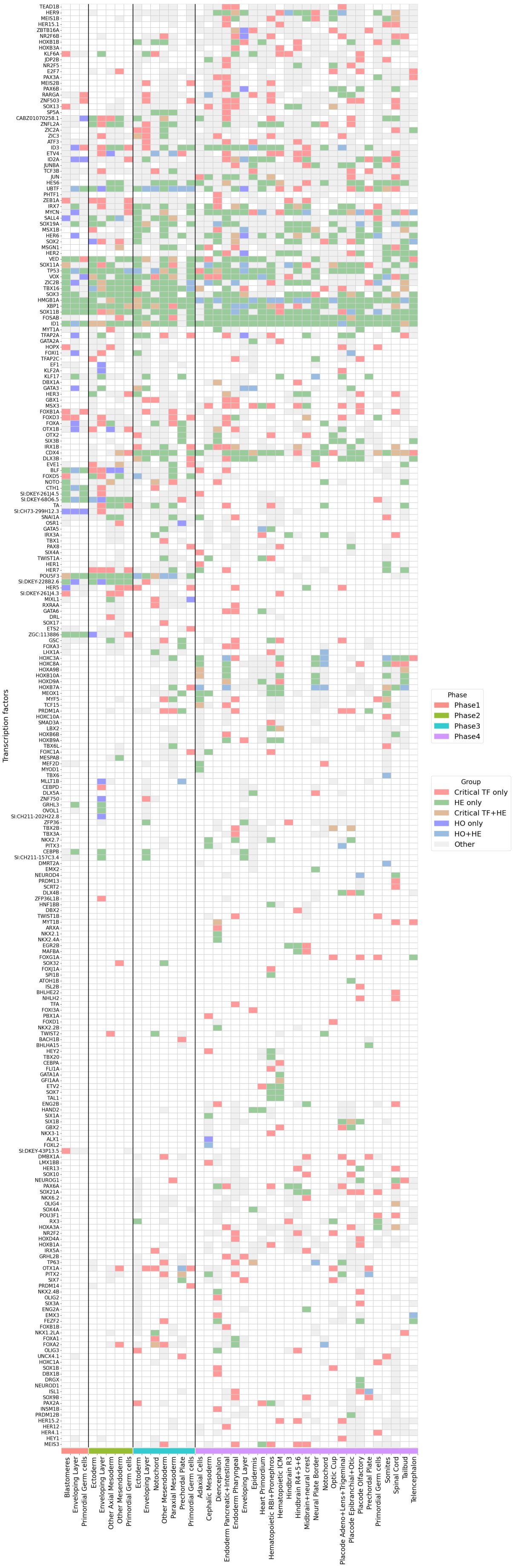

Supplementary Figure 8. TF category distribution across networks by developmental phase. Heatmap showing the distribution of transcription factor (TF) categories across the inferred gene regulatory networks (GRNs) spanning four developmental phases. Rows represent individual TFs, and columns correspond to GRNs ordered by phase. Each cell is colored according to the TF category identified in that network: red, Critical TF only; green, HE only; blue, Critical TF + HE; pink, HO only; purple, HO + HE; and gray, Other. Phase groups are indicated along the x-axis: Phase 1 (yellow), Phase 2 (green), Phase 3 (blue), and Phase 4 (purple). Vertical boundary lines separate GRNs belonging to different developmental trajectories.

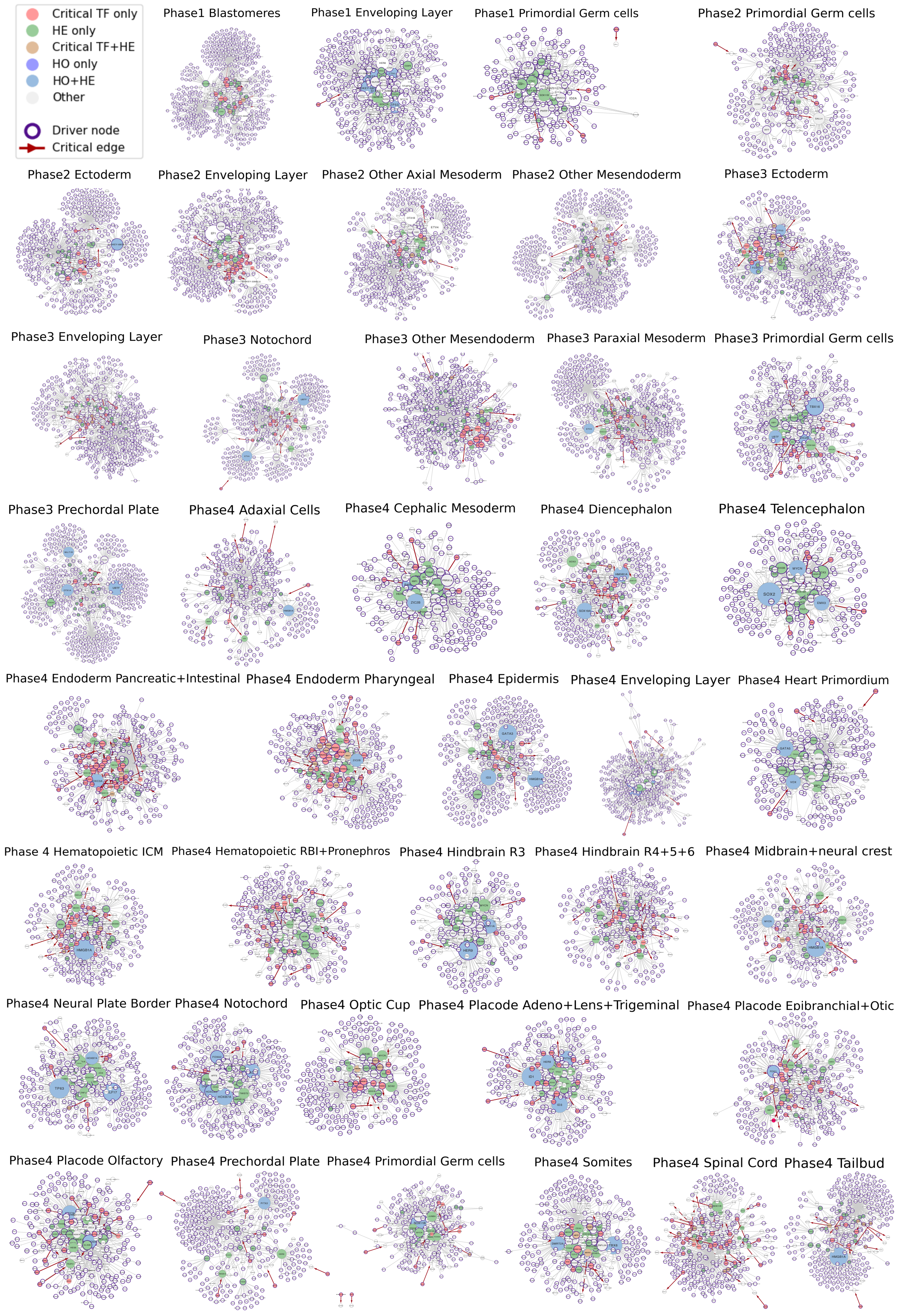

Supplementary Figure 9. Visualization of inferred gene regulatory networks (GRNs) across developmental phases and trajectories.

Each panel displays an inferred GRN corresponding to a single developmental trajectory across Phases 1–4. Nodes represent transcription factors (TFs) and target genes, colored according to TF category: red, Critical TF only; green, HE only; blue, Critical TF + HE; pink, HO only; purple, HO + HE; and gray, Other. Edges indicate regulatory interactions, with red edges marking critical edges. Nodes outlined with a purple halo denote driver nodes.
